# Genetic substructure in cynomolgus macaques (*Macaca fascicularis*) on the island of Mauritius

**DOI:** 10.1186/1471-2164-15-748

**Published:** 2014-08-31

**Authors:** Lisa M Ogawa, Eric J Vallender

**Affiliations:** New England Primate Research Center, Harvard Medical School, Southborough, MA 01772 USA

## Abstract

**Background:**

Nonhuman primates are commonly used in biomedical research as animal models of human disease and behavior. Compared to common rodent models, nonhuman primates are genetically, physiologically, behaviorally and neurologically more similar to humans owing to more recent shared ancestry and therefore provide the advantage of greater translational validity in preclinical studies. The cynomolgus macaque (*Macaca fascicularis*) is one of the most commonly used nonhuman primates in academic and industry settings, yet population genetic research has revealed significant substructure throughout the species distribution that may confound studies. Cynomolgus monkeys introduced to Mauritius specifically have previously been thought to maintain the least genetic heterogeneity of all cynomolgus monkeys, although recent work, including work from our lab, suggests macaques from Mauritius too may harbor cryptic substructure.

**Results:**

To evaluate putative substructure in Mauritian cynomolgus macaques, we designed a panel of 96 single nucleotide polymorphisms based on preliminary findings from previous work to screen 246 of cynomolgus monkeys from two primary suppliers. Results from this study support substructure in Mauritian macaques and suggest a minimum of two populations and maybe three on Mauritius, with moderate admixture.

**Conclusion:**

These findings inform the natural history of these monkeys suggesting either a previously unrecognized physical or ecological barrier to gene flow on Mauritius and/or the breakdown of historic substructure resulting from the history of macaque introduction to the island. These findings are relevant to ongoing research using these models in part because of increased appreciation of segregating common variation with functional effects and may be used to better inform animal selection in preclinical research.

**Electronic supplementary material:**

The online version of this article (doi:10.1186/1471-2164-15-748) contains supplementary material, which is available to authorized users.

## Background

Nonhuman primates are commonly used in scientific research as animal models of human behavior and disease. Although their use in research is overshadowed by the availability of less expensive and more abundant animal models such as rodents and zebrafish with easier husbandry, these animal models do not always provide the best translational medicine when trying to elucidate human pathologies. Nonhuman primates represent an alternative to these animal models and provide distinct advantages owing to their phylogenetic proximity to humans that lends itself to greater genetic, physiological, neurological, and behavioral similarities [1, 2]. Historically this has been most strongly recognized in pharmacokinetic studies [3, 4]. More recently, species differences have been pushed further with nonhuman primates showing increased translational validity in, for instance, regenerative medicine utilizing induced pluripotent stem cells [5, 6] and antiviral antibody therapy development [7, 8]. Macaque species are the most common nonhuman primate model, including the rhesus macaque (*Macaca mulatta*) and the long-tailed macaque (*M. fascicularis*), also commonly known as the crab-eating or cynomolgus macaque.

Despite a number of advantages to using nonhuman primates in biomedical research, there are distinct disadvantages that make studies in these animals difficult. Captive nonhuman primates harbor greater genetic heterogeneity, as these animals are outbred relative to laboratory strains of rodents, which is complicated by another distinct disadvantage which is that studies are often comprised of much smaller sample sizes inherent in the increased costs required for acquisition and husbandry [1]. Because it has become increasingly apparent in humans, and to a lesser extent macaques, that small changes in a gene or regulatory region can have large effects on the function of that gene, better information regarding genetic variation in these research models has become a necessity. The effects of genetic stratification or cryptic population substructure on studies, particularly where samples sizes are small, are significant [9]. In macaques, population differences have been identified with regards to behavior [10], physiology [11], susceptibility to infectious disease [12], and toxicology [13]. Therefore a priori genetic information on nonhuman primates used in research can aid in more informed selection of individuals for studies and better translational models.

The cynomolgus macaque is one of the most commonly used models in biomedical research [14, 15]. These animals are particularly beneficial in common studies relating to toxicology and preclinical therapy development in which the closer ancestry of nonhuman primates to humans is desired [16–18]. Furthermore cynomolgus macaques specifically from the island of Mauritius may provide the best alternative to Indian origin rhesus macaques for studying HIV/AIDS based on a high level of MHC class I allele sharing [19], and may be the only nonhuman primate animals for the study of hepatitis B virus as the virus is naturally occurring and transmissible only in this population of macaques [20].

Cynomolgus macaques originated in Southeast Asia and maintain a wide subtropical distribution ranging from Vietnam, Cambodia, Thailand and Malaysia to island populations in Indonesia, the Philippines and, more recently, Mauritius. Animals used in research are sourced from many of these locations and, while subpopulation differences are still minimally studied, potentially important genetic differences between populations are already recognized [21, 22]. Mauritian macaques are attractive for biomedical research for a number of reasons. One is their putative genetic homogeneity. The history of cynomolgus macaques on Mauritius is largely undocumented, but many believe they arrived along with Portuguese or Dutch sailors sometime in the 16^th^ century [23]. Recent molecular work suggests these macaques derive from individuals from Java, Indonesia [24] or more likely Sumatra, Indonesia [25]. Cynomolgus macaques are broad generalists like their rhesus macaque sister species [26]. On the island of Mauritius these macaques primarily exploit the human disturbed habitats and are an invasive species and a disruptive “pest” [23, 27].

With many cynomolgus macaques used in research sourced from Mauritius, here we utilized a pseudogenomic approach to investigate population homogeneity in this group of macaques. Although previous work has found little genetic heterogeneity [24], recent work from our lab [28] and others [29] suggests population substructure in Mauritian macaques that may have been missed. This study was therefore designed to follow-up on preliminary findings in the Goswami et al. [28] study and to utilize single nucleotide polymorphisms (SNPs) from the study to develop a panel that differentiates between the two putative populations to screen a larger sample size. Population genetic structure in Mauritian cynomolgus macaques would be significant given the limitations of nonhuman primate research and would suggest that better care should be given to the selection of these animals for translational scientific research.

## Results and discussion

Previous work [28] found a signature of genetic substructure in 32 unrelated cynomolgus macaques on the island of Mauritius. Because the original study was not designed to detect population substructure, the variation it identified was not entirely evolutionarily neutral nor independent. While initial studies using bootstrapping showed the substructure to be robust, it remained unclear if there was a cryptic artifactual source. Because substructure between geographically disparate cynomolgus macaque groups is well-established (i.e. Southeast Asian cynomolgus monkeys are genetically distinguishable from those in Indonesia and the Philippines) [21, 22], we first evaluated whether animals from the initial study were in fact Mauritian-derived.

**Figure 1:**
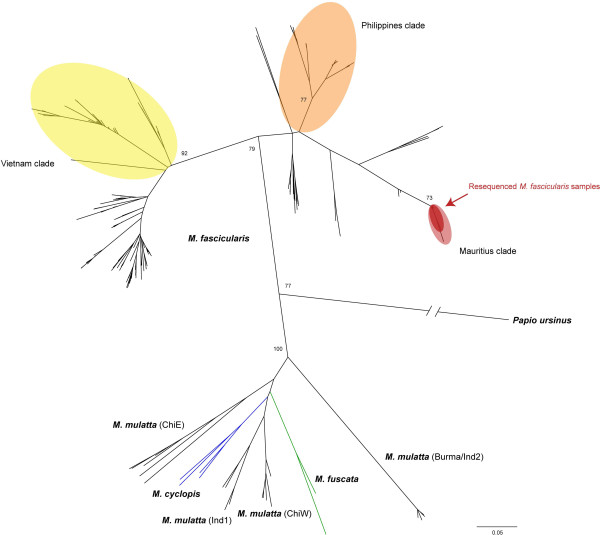
**mtDNA phylogeny of macaques.** Maximum likelihood phylogenetic tree derived from a ~835 bp segment of the cytochrome b gene to confirm provenance of cynomolgus macaques in the original Goswami et al. [28] study. Animals included in the analysis are referenced in Additional file 1: Table S1. Notable macaque clades are highlighted according to previous work on the subject [46]. Animals from Goswami et al. [28] are highlighted in dark red and fall wholly within the Mauritian cynomolgus macaque group, supporting that they derived from Mauritius.

Using mitochondrial (mtDNA) and Y-chromosome DNA (YDNA), we tested whether the 32 animals from the Goswami et al. study [28] clustered with known Mauritius-origin animals or with cynomolgus macaque populations from other parts of the species range including Southeast Asia, the Philippines and Indonesia. Phylogenetic analysis of mtDNA of 516 individuals (484 from previous studies plus the 32 in question; Additional file 1: Table S1) revealed 344 variable sites overall and 273 that were parsimony informative. From 31 of the 32 cynomolgus macaques in the initial study we obtained 794 bp of mtDNA sequence data and from the last 721 bp. Consistent with previous studies of mtDNA variation in macaques our phylogenetic analysis distinguishes with high support cynomolgus macaques as a monophyletic clade distinct from *M. mulatta*, *M. cyclopis*, and *M. fuscata* (Figure 1). This analysis also distinguishes a Vietnam clade, Philippines clade and Mauritius clade of cynomolgus macaques, with 28 of the initial subset of animals sharing a single haplotype, 4 differing from the common haplotype by a single nucleotide, and one with 8 unique singleton mutations, and all falling within the Mauritius clade.

**Figure 2:**
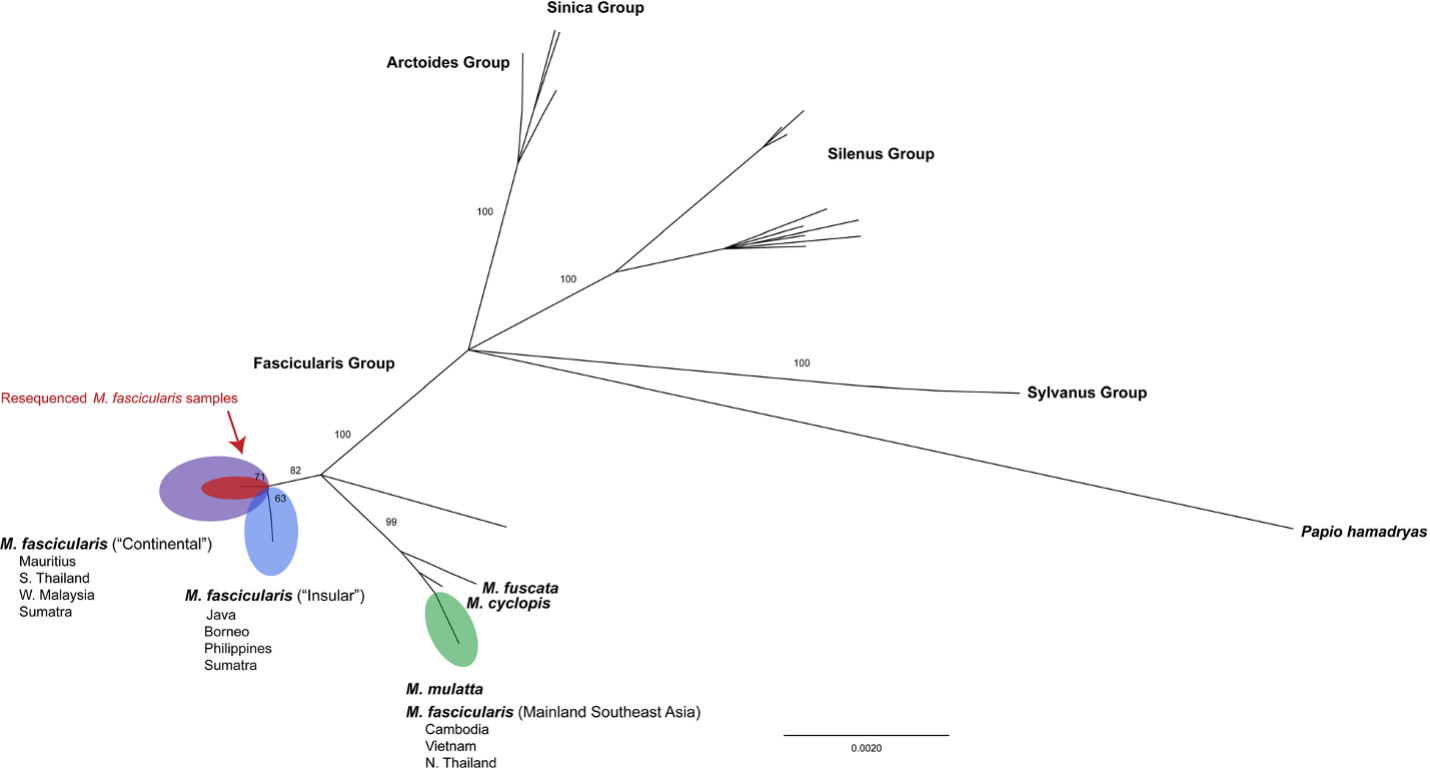
**Y-chromosome phylogeny of macaques.** Maximum likelihood phylogenetic tree derived from ~2850 bp of the *SRY* and *TSPY* genes to confirm provenance of cynomolgus macaques in the original Goswami et al. [28] study. Notable clades are highlighted according to previous work on the subject [25, 44] and additional macaque groups are identified based on Tosi et al. [44] and defined in supplemental Additional file 2: Table S2, including the monospecific Arctoides (*M. arctoides*) and Sylvanus (*M. sylvanus*) groups, the Sinica group, and the Silenus group. Animals from Goswami et al. [28] are highlighted in red and fall within the “Continental” clade of cynomolgus macaques that includes those from Mauritius, supporting that they derived from Mauritius.

Analysis of the YDNA revealed similar findings. Combined *SRY* and *TSPY* sequence data of 102 individuals (70 from previous studies plus the 32 in question; Additional file 2: Table S2) revealed 135 variable sites overall and 77 that were parsimony informative. From the 32 cynomolgus macaques we obtained 612 bp of *SRY* sequence data from each individual, and 2172 bp of *TSPY* sequence data from 28 of the 32 individuals. For the rest at least 97.5% of the full sequence length was covered. Again consistent with previous studies, our phylogenetic analysis distinguishes the fascicularis group of macaques from other macaque species, with *M. fuscata* and *M. cyclopis* clustering with a clade of cynomolgus macaques from mainland Southeast Asia as well as *M. mulatta* (Figure 2). Our analysis also distinguishes few but consistent mutations between the “Continental” and “Insular” clades (defined as Thailand/Cambodia/Vietnam/Western Malaysia and Sumatra/Java/Borneo/Philippines respectively) of cynomolgus macaques with the initial subset of animals clustering with other Mauritius individuals in the “Continental” clade (bootstrap value = 70), represented by only a single haplotype.

These mitochondrial and Y chromosome DNA analyses confirm that the initial subset of animals were indeed of Mauritius-origin and that the genetic substructure identified is relevant to the island and not of a more general origin. It remains, however, that this initial study was not conceived as a population genetics, but rather as a functional genetics, study. The variation that was used in this initial analysis was not independent, was not entirely randomly distributed across the genome, and was not necessarily selectively neutral. In order to test the generalizability of the population substructure we sought to replicate the findings using a much larger pool of Mauritian-origin cynomolgus macaques with specific SNPs chosen that would be more suitable to population genetic studies.

Although we acknowledge the ascertainment bias, to further test the validity of these data we created a targeted SNP panel of selected SNPs from the Goswami et al. [28] study prioritizing SNPs with the greatest differences in minor allele frequencies (ΔMAF) between the two putative populations, an approach utilized previously in the literature for similar research [30–32]. SNPs were further selected to be distributed relatively evenly across the 20 autosomes and were >150 kb apart (median distance between adjacent SNP pairs of 11 Mb) to enhance selection of independently inherited mutations and limiting selection of SNPs in physical linkage [33]. Particular attention was also paid to selection of SNPs in selectively neutral regions although given the scope of the original paper, loci in untranslated regions (UTRs) and coding regions could not be avoided and these are likely in regions that are under purifying selection. Of the 110 assays designed to target the selected SNP loci, 4 failed, 3 were deemed low quality assays, and 5 had call rates below our 95% cutoff level. An additional 2 were apparently monomorphic. It is unclear if these represent technical failures or identification failures. Regardless, all these assays were excluded from the analysis. A total of 96 loci were therefore analyzed on additional cynomolgus macaques acquired from two primary sources of Mauritius-origin cynomolgus monkeys (Bioculture Mauritius and Cynologics via Primate Products) for a total of 246 individuals, including the 32 individuals in the original study.

**Figure 3:**
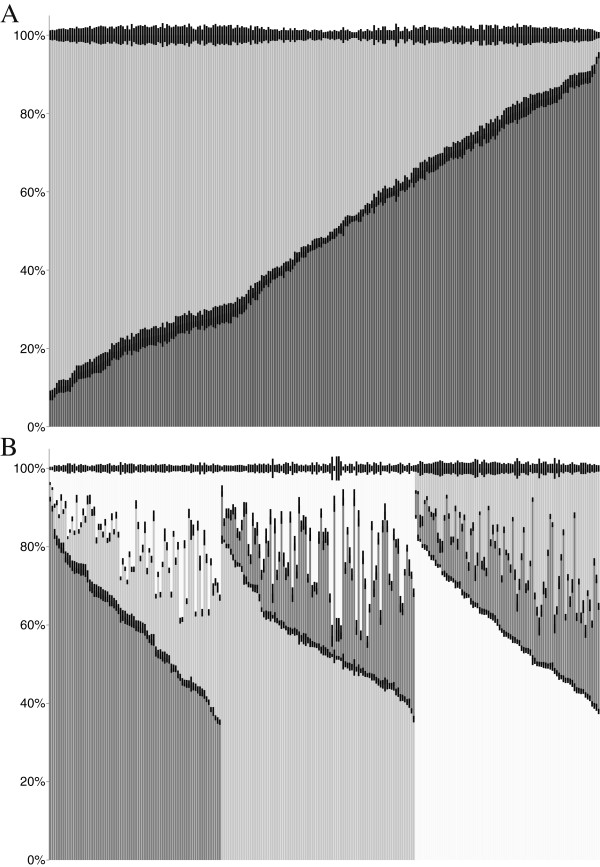
**Inferred ancestry of individuals from STRUCTURE. A**. Inferred ancestry with K = 2. **B**. Inferred ancestry with K = 3. Animals are arranged in decreasing proportion of their predominant subpopulation.

STRUCTURE analysis of the SNP data on the 246 individuals supports the two population model suggested by Goswami et al. [28]; however, this dataset also suggests the possibility that three populations are represented by the data (Figure 3). The “correct” or “true” number of clusters has traditionally been identified by identifying the maximal value of log likelihood value, however, it has been established that log likelihood will plateau or increase incrementally at higher values of K once the “true” value is reached [34]. A difficulty lies in identifying at what point this plateau begins. Evanno et al. [34] proposes using the modal value of ΔK, the second order rate of change divided by the standard deviation. Here, both approaches are presented.

**Figure 4:**
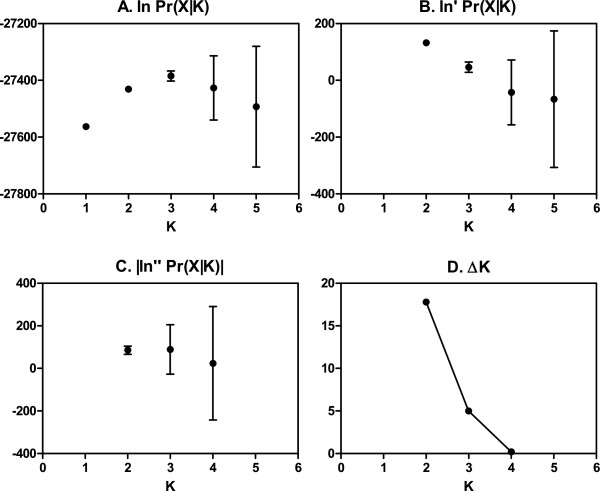
**Subpopulation inference after Evanno et al.** [34]**, default STRUCTURE model with a 10 k burn-in and 40 k MCMC replicates. A**. Mean ln (X|K) (±SD) over 100 runs for each value of K. **B**. Rate of change of ln (X|K) (mean ± SD). **C**. Absolute value of the second order rate of change (mean ± SD). **D**. ΔK, mean of ln“(X|K) divided by standard deviation of ln (X|K). The modal value is the presumptive true number of subpopulation.

The first approach used the informal guidelines outlined for STRUCTURE v2.3 [35]. One hundred independent runs assuming admixture and correlated allele frequencies (λ = 1) with an MCMC length of 40,000 (10,000 burn-in) converged on a K of 3 (Fst = 0.11, 0.07, 0.06), where 3 is the smallest value of K when ln Pr (X|K) plateaus. Second, we used the more formal approach outlined by Evanno et al. [34]. This approach suggests the “true K” exists where the second order rate of change of ln Pr (X|K) with respect to K (ΔK) is the greatest; in our dataset, this occurs at a K of 2 (Fst = 0.08, 0.03) (Figure 4). Both interpretations of the dataset were consistent when estimating lambda (λ = 2.22) and when the MCMC length was increased to 250,000 (50,000 burn-in) (Additional file 3: Figures S1 and Additional file 4: Figure S2). Given that the correlated allele frequencies model has the potential to overestimate K [36], data was also run using the independent model even though it may not be appropriate for these data, and results did not change (Additional file 5: Figure S3). It is therefore conservative to infer two subpopulations of cynomolgus macaques exist on the island of Mauritius, but these data do not exclude a three subpopulation model.

Following these analyses, we were then able to revisit our SNP panel. For each SNP the minor allele frequencies in each subpopulation were determined from the STRUCTURE analysis and we were able to recalculate ΔMAF (Table 1). Perhaps as expected we saw a number of SNPs that did not differentiate between the subpopulations. For K of 2; 9 had ΔMAF values less than 1% and 14 less than 2%. Nevertheless, the overall median ΔMAF for the 96 SNPs was 8.4% and 40 of the 96 had ΔMAF values greater than 10%. For K of 3, only 2 SNPs do not show a ΔMAF greater than 2% for at least one pair of subpopulations. The median maximum ΔMAF is 15.2%, while the median pairwise ΔMAF are 12.1%, 8.8%, and 7.2%. We also examined whether ΔMAF values were correlated with chromosome and they are not (data not shown), or SNP position relative to gene, and again they are not (data not shown). We are confident then that while this SNP panel may not be of maximal power, it is free of artifactual biases.

**Table 1:** **SNPs with minor allele frequencies (MAF) determined through STRUCTURE analysis**

		K = 2			K = 3							
Chromosome	Position	Allele 1	Allele 2	MAF* Pop1	MAF* Pop2	ΔMAF	MAF* Pop1	MAF* Pop2	MAF* Pop3	ΔMAF (1,2)	ΔMAF (1,3)	ΔMAF (2,3)
chr1	9589467	G	A	0.154	0.272	0.117	0.146	0.198	0.298	0.052	0.152	0.100
chr1	62765400	C	T	0.265	0.357	0.092	0.190	0.483	0.254	0.293	0.063	0.230
chr1	112321563	C	T	0.530	0.417	0.112	0.547	0.496	0.376	0.05	0.171	0.120
chr1	114484199	G	A	0.290	0.202	0.088	0.261	0.326	0.147	0.065	0.114	0.179
*chr1*	*128959247*	*C*	*G*	*0.367*	*0.359*	*0.008*	*0.363*	*0.436*	*0.288*	*0.073*	*0.075*	*0.148*
chr1	197826522	G	A	0.361	0.261	0.101	0.425	0.216	0.293	0.210	0.132	0.078
chr1	215456600	A	G	0.164	0.208	0.044	0.156	0.206	0.198	0.051	0.042	0.008
chr2	20855998	T	C	0.292	0.342	0.050	0.272	0.395	0.283	0.123	0.011	0.112
chr2	34182166	G	C	0.351	0.498	0.147	0.262	0.627	0.381	0.366	0.119	0.246
chr2	87829723	G	A	0.256	0.085	0.172	0.337	0.117	0.055	0.221	0.282	0.062
chr2	90581348	T	C	0.290	0.169	0.121	0.349	0.167	0.173	0.181	0.176	0.005
chr2	97192885	G	A	0.180	0.154	0.026	0.195	0.096	0.209	0.100	0.014	0.113
chr2	133230027	A	T	0.388	0.341	0.046	0.413	0.304	0.376	0.109	0.037	0.072
*chr2*	*134823729*	*T*	*C*	*0.338*	*0.352*	*0.014*	*0.363*	*0.278*	*0.397*	*0.085*	*0.034*	*0.120*
*chr2*	*136472042*	*G*	*T*	*0.118*	*0.116*	*0.002*	*0.150*	*0.049*	*0.152*	*0.101*	*0.003*	*0.103*
*chr3*	*95549222*	*T*	*C*	*0.469*	*0.470*	*0.001*	*0.471*	*0.478*	*0.461*	*0.007*	*0.010*	*0.017*
chr3	164081759	A	G	0.215	0.336	0.121	0.168	0.358	0.300	0.190	0.132	0.058
chr3	192188304	A	G	0.441	0.404	0.037	0.434	0.414	0.418	0.020	0.016	0.004
chr4	46479393	G	A	0.150	0.571	0.421	0.118	0.211	0.746	0.093	0.628	0.535
chr4	46834407	C	T	0.189	0.571	0.382	0.163	0.207	0.765	0.044	0.602	0.559
chr4	47617528	A	C	0.487	0.272	0.215	0.528	0.419	0.189	0.109	0.338	0.229
chr4	116987652	T	G	0.209	0.324	0.115	0.178	0.330	0.295	0.152	0.117	0.035
chr4	131253869	C	T	0.417	0.384	0.033	0.388	0.429	0.382	0.041	0.006	0.047
chr5	96690554	C	T	0.177	0.263	0.085	0.134	0.291	0.234	0.157	0.100	0.057
chr5	178329879	T	C	0.149	0.316	0.167	0.122	0.326	0.251	0.204	0.128	0.076
chr6	72896506	T	C	0.468	0.266	0.202	0.507	0.309	0.283	0.198	0.224	0.026
chr6	86863653	C	T	0.414	0.347	0.067	0.463	0.334	0.346	0.128	0.116	0.012
chr6	145047256	C	A	0.132	0.240	0.108	0.107	0.203	0.250	0.096	0.143	0.047
chr7	30401169	C	G	0.351	0.375	0.024	0.348	0.407	0.335	0.059	0.013	0.072
chr7	53553308	C	T	0.427	0.209	0.219	0.468	0.263	0.222	0.205	0.246	0.042
*chr7*	*59074588*	*T*	*G*	*0.147*	*0.145*	*0.002*	*0.155*	*0.133*	*0.151*	*0.022*	*0.004*	*0.018*
chr7	87206195	T	A	0.410	0.322	0.088	0.436	0.355	0.305	0.081	0.131	0.050
chr7	101174441	G	C	0.272	0.309	0.037	0.262	0.246	0.364	0.015	0.102	0.118
chr7	144144421	G	C	0.456	0.357	0.099	0.487	0.301	0.432	0.186	0.055	0.131
chr7	154450353	G	T	0.521	0.438	0.083	0.551	0.413	0.473	0.138	0.078	0.060
chr7	162987878	G	A	0.231	0.280	0.049	0.178	0.317	0.269	0.139	0.091	0.049
chr7	168414331	C	T	0.164	0.136	0.028	0.189	0.158	0.105	0.031	0.084	0.053
chr8	28713522	G	A	0.294	0.241	0.053	0.301	0.246	0.255	0.055	0.046	0.009
chr8	38422084	C	T	0.319	0.276	0.044	0.337	0.220	0.336	0.118	0.001	0.116
*chr8*	*55827375*	*G*	*A*	*0.331*	*0.326*	*0.005*	*0.293*	*0.293*	*0.401*	*0.001*	*0.108*	*0.107*
chr8	67208693	A	G	0.259	0.471	0.212	0.193	0.446	0.459	0.253	0.267	0.013
chr8	143859237	G	A	0.153	0.193	0.040	0.111	0.262	0.143	0.151	0.032	0.119
chr9	25787241	A	G	0.187	0.229	0.042	0.189	0.180	0.258	0.009	0.069	0.077
chr9	90272749	T	C	0.054	0.268	0.214	0.030	0.265	0.192	0.235	0.162	0.073
chr9	132778054	T	C	0.361	0.513	0.151	0.299	0.549	0.463	0.251	0.164	0.086
chr10	10319647	C	G	0.510	0.385	0.125	0.538	0.408	0.395	0.130	0.142	0.013
chr10	38608009	G	A	0.144	0.271	0.127	0.119	0.319	0.185	0.200	0.066	0.133
chr10	87110837	C	T	0.293	0.153	0.140	0.316	0.251	0.101	0.065	0.215	0.150
chr10	90566608	A	G	0.131	0.243	0.112	0.085	0.304	0.170	0.219	0.086	0.134
chr11	7005631	C	G	0.275	0.531	0.256	0.190	0.519	0.503	0.329	0.313	0.016
chr11	13375736	G	T	0.147	0.127	0.020	0.167	0.087	0.159	0.080	0.008	0.072
chr11	99848243	G	A	0.195	0.396	0.202	0.146	0.412	0.333	0.266	0.186	0.079
chr11	123966677	C	T	0.384	0.467	0.083	0.380	0.416	0.482	0.036	0.102	0.066
chr12	94765925	G	A	0.600	0.307	0.294	0.672	0.405	0.280	0.267	0.392	0.125
chr12	94964954	C	G	0.180	0.418	0.237	0.136	0.291	0.475	0.156	0.339	0.183
*chr12*	*100440950*	*G*	*A*	*0.234*	*0.220*	*0.014*	*0.240*	*0.230*	*0.212*	*0.010*	*0.027*	*0.017*
chr13	49122594	G	A	0.282	0.362	0.080	0.233	0.370	0.364	0.137	0.130	0.007
chr13	125692849	C	G	0.251	0.420	0.169	0.181	0.468	0.356	0.287	0.175	0.112
chr13	133631999	A	G	0.234	0.348	0.114	0.205	0.399	0.269	0.194	0.064	0.130
chr13	134111985	G	A	0.155	0.084	0.071	0.188	0.073	0.098	0.115	0.090	0.025
chr14	451418	C	T	0.179	0.302	0.123	0.135	0.378	0.210	0.243	0.075	0.167
chr14	3231322	A	G	0.220	0.253	0.033	0.219	0.202	0.290	0.017	0.071	0.088
chr14	10137714	A	G	0.233	0.160	0.074	0.296	0.134	0.160	0.162	0.136	0.025
chr14	44393953	A	G	0.138	0.222	0.085	0.091	0.274	0.175	0.183	0.084	0.099
chr14	52927439	G	A	0.442	0.503	0.060	0.435	0.497	0.487	0.062	0.052	0.010
*chr14*	*57078000*	*A*	*G*	*0.395*	*0.379*	*0.016*	*0.424*	*0.370*	*0.369*	*0.054*	*0.055*	*0.002*
chr14	65934862	C	A	0.304	0.196	0.108	0.373	0.147	0.229	0.226	0.145	0.082
chr14	69046745	G	A	0.233	0.062	0.172	0.327	0.048	0.064	0.280	0.263	0.017
chr14	71412148	C	T	0.404	0.220	0.184	0.513	0.169	0.254	0.344	0.259	0.085
chr14	91544113	C	T	0.407	0.493	0.086	0.338	0.494	0.517	0.157	0.179	0.023
chr14	103525965	G	A	0.052	0.202	0.150	0.037	0.173	0.175	0.136	0.138	0.002
*chr14*	*117290848*	*C*	*T*	*0.301*	*0.293*	*0.009*	*0.340*	*0.188*	*0.364*	*0.152*	*0.023*	*0.175*
*chr15*	*8694018*	*C*	*G*	*0.390*	*0.388*	*0.002*	*0.395*	*0.371*	*0.401*	*0.024*	*0.006*	*0.030*
chr15	37923113	C	T	0.395	0.381	0.015	0.420	0.313	0.432	0.107	0.012	0.119
*chr15*	*85452749*	*T*	*C*	*0.452*	*0.442*	*0.009*	*0.471*	*0.400*	*0.469*	*0.071*	*0.002*	*0.069*
chr16	69710848	G	A	0.184	0.216	0.032	0.175	0.260	0.166	0.085	0.009	0.094
chr16	77232074	C	T	0.187	0.319	0.132	0.113	0.238	0.405	0.124	0.292	0.168
chr16	77856955	C	T	0.357	0.432	0.076	0.269	0.379	0.534	0.110	0.265	0.155
chr17	77388581	A	G	0.254	0.226	0.028	0.274	0.146	0.299	0.128	0.025	0.154
*chr17*	*79668813*	*G*	*A*	*0.440*	*0.426*	*0.014*	*0.444*	*0.429*	*0.426*	*0.015*	*0.018*	*0.003*
chr18	53659440	G	A	0.509	0.463	0.046	0.507	0.451	0.500	0.055	0.006	0.049
chr18	70696212	C	A	0.124	0.171	0.048	0.096	0.168	0.179	0.072	0.083	0.011
chr19	3035008	G	A	0.229	0.269	0.040	0.207	0.291	0.248	0.084	0.042	0.043
chr19	6802211	T	C	0.164	0.188	0.024	0.118	0.277	0.131	0.159	0.013	0.146
chr19	9916169	G	A	0.445	0.474	0.029	0.413	0.449	0.514	0.036	0.102	0.065
chr19	13840476	G	A	0.127	0.214	0.087	0.076	0.308	0.126	0.232	0.050	0.182
chr19	14183472	G	A	0.519	0.206	0.313	0.523	0.468	0.090	0.055	0.432	0.377
chr19	14337213	T	C	0.472	0.350	0.122	0.496	0.269	0.469	0.227	0.027	0.200
chr19	15160971	A	C	0.325	0.509	0.184	0.302	0.486	0.466	0.184	0.164	0.020
chr19	47366874	G	A	0.183	0.230	0.048	0.175	0.189	0.257	0.014	0.082	0.068
chr19	52125587	C	T	0.489	0.386	0.104	0.532	0.405	0.375	0.127	0.157	0.030
chr19	52960498	G	A	0.287	0.198	0.089	0.358	0.131	0.239	0.227	0.119	0.108
chr19	53650389	C	G	0.397	0.421	0.024	0.364	0.412	0.451	0.047	0.086	0.039
chr20	18839854	T	C	0.203	0.330	0.126	0.172	0.376	0.252	0.204	0.080	0.124
chr20	19030347	A	G	0.412	0.466	0.054	0.400	0.451	0.467	0.051	0.067	0.016
*chr20*	*56038933*	*C*	*T*	*0.073*	*0.078*	*0.005*	*0.079*	*0.107*	*0.041*	*0.028*	*0.038*	*0.066*

Data for two (K = 2) and three (K = 3) subpopulations is shown. Some SNPs failed to differentiate between the subpopulations and are emphasized; given a K of 2, ΔMAF values less than 1% are highlighted in dark grey and ΔMAF values between 1% and 2% are highlighted in light grey. Minor allele designation is made relative to the population as a whole; some subpopulations may have minor allele frequencies greater than 0.5. ΔMAF: Difference in minor allele frequencies between subpopulations.

**Figure 5:**
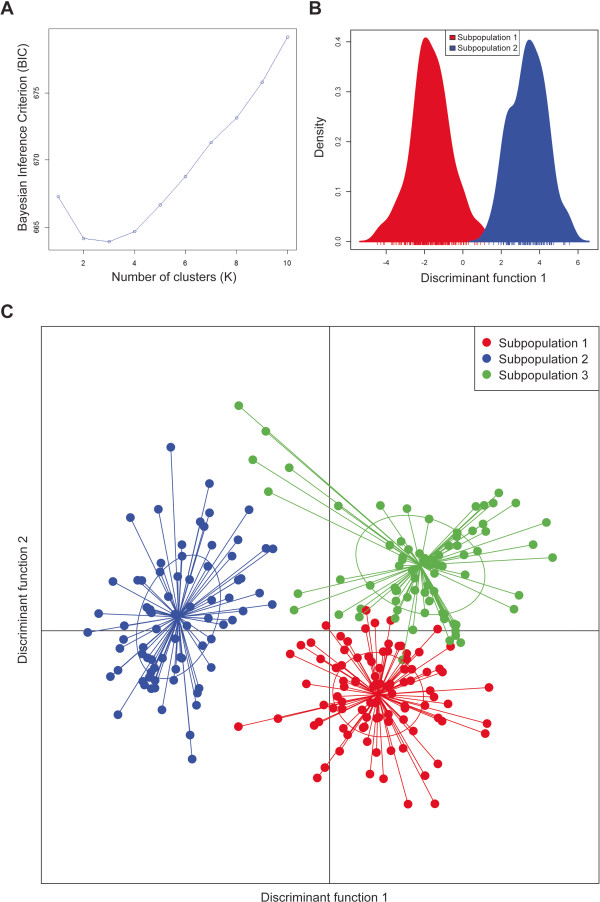
**Discriminant Analysis of Principal Components (DAPC) to infer population substructure. A**. Bayesian Inference Criterion (BIC) values versus number of clusters (K) **B**. Inferred ancestry with K = 2 **C**. Inferred ancestry with K = 3. Results support STRUCTURE analysis findings that suggest the data are best represented by two or three subpopulations. This analysis however suggests three subpopulations may be more likely than two given the lower BIC value when K = 3.

To confirm the findings of the STRUCTURE analysis, a second approach, Discriminant Analysis of Principal Components (DAPC), was also performed to analyze the data. This methodology uses an approach conceptually similar to Principal Component Analysis (PCA) with a focus on minimizing sources of within group variation [37]. *k*-means clustering on principal components derived from allele frequencies we compared using Bayesian Information Criteria (BIC) (Figure 5A). As with STRUCTURE, results suggest two or three subgroups with *k*-means clustering slightly favoring the latter. Using DAPC with either K of 2 (Figure 5B) or K of 3 (Figure 5C) it is possible to visualize membership within these groups. Though there is a real question of overfitting of the data with DAPC, it is clear that the findings of STRUCTURE hold up across different methodologies.

Regardless of whether there are two or three subpopulations, moderate admixture (mean alpha in STRUCTURE for K of 2 = 0.84, K of 3 = 0.68) is observed and suggests either reproductively isolated populations with hybrid zones or historic isolation that no longer exists. Previous work on Mauritian cynomolgus macaques has suggested genetic substructure with a putative northern and southern population based on microsatellite loci [29]. Unfortunately capture location data on all the individuals in this study could not be acquired and therefore it is difficult to evaluate whether we see a similar pattern. Capture location data provided by Bioculture Mauritius/Charles River Laboratories however on the original subset of 32 macaques does not support an obvious physical barrier to gene flow or geographic pattern (data not shown), but does not exclude the potential for an unrecognized ecological “barrier.” These data further do not support population differences based on source/dealer; cynomolgus monkeys from both Cynologics Ltd. and Bioculture Mauritius Ltd. are represented in each of the subpopulations (Additional file 6: Figure S4).

Importantly, it seems that despite this population substructure most animals are significantly admixed and the structure may be decaying. Unlike other subpopulations of cynomolgus macaques, such as those from the various “continental” and “insular” location, or the Indian-origin and Chinese-origin rhesus, the two subpopulations of Mauritian macaques appear largely admixed. This can be envisioned similarly to the Collaborative Cross of laboratory mouse strains [38], Chinese-Indian hybrid colonies of rhesus macaques [31], or even admixed human populations such as African-Americans [39] or Latinos [40]. Admixed populations can offer demographic challenges similar to other sources of cryptic population substructure, but can also be important in the mapping of quantitative traits [41]. In the preclinical studies where Mauritian cynomolgus macaques are often used, this can mean both that there may be cryptic genetic effects on phenotypes of study, potentially confounding experimental and control groups, and that it may be more straightforward to identify functional genetic variation associated with the phenotypes. To do so would, naturally, require a higher density of genetic variation than we have made use of here, but with next generation sequencing costs becoming increasingly more tractable, this is perhaps a fertile area for future study.

The underlying cause of this apparent population stratification remains unknown. An ecological separation remains possible, but there is little evidence to support this hypothesis and the likelihood of it causing pervasive genome wide effects in such a relatively short time seems minimal. What may be more likely given the relatively recent introduction of macaques to Mauritius by mariners is that these data may suggest either two or three independent founding events and/or, knowing that significant genetic structure exists among cynomolgus macaques outside of Mauritius [21], founding individuals derived from two or three different locales in their native range. It is also possible that these data simply reflect more traditional founder effects, particularly if effective founding populations among extant animals were lower than previously appreciated.

## Conclusion

Animal models are an important component of preclinical biomedical research and critical to the translational success of new drugs and therapies. Just as the importance of cryptic substructure in human studies became increasingly recognized, so too now do we recognize its importance in animal models. In laboratory mice and rats, animals are almost always inbred and genetically identical, but non-human primates are outbred and can harbor genetic variation often exceeding that seen in humans. Moreover, an increasing number of studies have identified segregating common variation with explicit functional effects on many of the characteristics under study in these animals. It is important, therefore, that the genetic substructure of populations be taken into account.

Cynomolgus macaques are among the most widely used of non-human primate model species, particularly in industry, and among cynomolgus macaques those sourced from Mauritius are particularly prevalent. Moving into the post-genomic era, it has become increasingly possible to tease apart the complex demographic history and extant genetic difference among this population. Although there are no obvious or otherwise documented phenotypic (i.e. physiological, biological, ecological) differences among these subpopulations of cynomolgus macaques on the island of Mauritius, given the results of our study it is entirely possible that observed phenotypic variability among Mauritian cynomolgus macaques [11, 42, 43] may accompany the genetic substructure. And while the substructure does not appear to be as stark or as significant as that between, for instance, Chinese- and Indian-origin rhesus macaques or subpopulations of cynomolgus macaques from Vietnam, Indonesia, and the Philippines, it has nevertheless aroused interest and investigation into these differences would be a meaningful avenue of future research.

Here we demonstrate at least two, and possibly three, subpopulations of cynomolgus macaques on Mauritius. We propose that this stratification is suggestive of either an unknown and previously unrecognized barrier to gene flow, or the remains of genetic substructure among Mauritian cynomolgus macaques following distinct founding events and/or founding individuals approximately 500 years ago. This effort has served to not only elucidate the natural history of cynomolgus macaques on Mauritius, but to also better inform studies that use these animals.

## Methods

### Ethics statement

Blood draws for the isolation of genomic DNA for animals housed at the NEPRC were done during routine preventative health care by trained veterinary phlebotomists within the NEPRC Division of Veterinary Resources. All animals were maintained in accordance with the guidelines of the Harvard Medical School Standing Committee on Animals and the Guide for Care and Use of Laboratory Animals of the Institute of Laboratory Animal Resources, National Research Council. Blood draws from animals obtained from Cynologics Ltd. (Port Louis, Mauritius) and Bioculture Mauritius Ltd. (Senneville, Maurtitus) were collected at the respective sources in accordance with their standard operating procedures.

### Sample collection

The two primary suppliers of cynomolgus macaques from Mauritius are Bioculture Mauritius and Cynologics. These animals are captured and subsequently resold or bred by secondary distributors such as Charles River Laboratories (Wilmington, MA) and Primate Products (Miami, FL). The 32 cynomolgus macaque genomic DNAs used in the Goswami et al. [28] study were acquired from animals housed at the New England Primate Research Center, originally purchased from Charles River Laboratories, and ultimately derived from Bioculture Mauritius. In addition to these animals, we obtained cynomolgus macaque whole blood from Cynologics (78 samples) and Bioculture Mauritius (135 samples), both directly and through U.S. distributors of their animals. Because these animals were wild-caught information on relatedness among animals is unknown, however animals were derived from numerous capture sites across Mauritius and from distinct troops. Genomic DNA from the animals was isolated from 1–3 mL of whole blood using the FlexiGene DNA Kit (Qiagen, Valencia, CA).

### mtDNA and Y-chromosome ancestry

PCR, sequencing and analysis of the mtDNA and Y chromosome were performed on the 32 Goswami et al. [28] cynomolgus macaques to verify Mauritius origin (Table 2). Regions selected for amplification and sequencing include previous regions utilized to evaluate the phylogenetics and biogeography of cynomolgus macaques [44–46]. The mtDNA region targeted was a ~835 base pair (bp) portion of the d-loop including the first hypervariable segment (HSV I) and part of the cytochrome b gene. This region was amplified using primers from Smith and McDonough [45] in a 25 μl reaction using GoTaq Green Master Mix (Promega, Madison, WI), 50 ng DNA, and a thermal cycling protocol that included an initial 2 min denaturation step at 95°C, 40 cycles of 95°C for 30 s, 63°C for 30 s, 72°C for 90 s, and a final elongation step at 72°C for 7 min. On the Y chromosome, the *SRY* (sex-determining region) and *TSPY* (testis-specific protein) genes were targeted for amplification and analysis. The *SRY* gene (~600 bp) was amplified using primers SW2 and SW3B from Whitfield et al. [47] and the same PCR and thermal cycling protocol as above was used with an annealing temperature of 60°C rather 63°C. The *TSPY* gene (~2250 bp) was amplified using primers TSPY-A and TSPY5R from Tosi et al. [44]. Due to the longer target size, amplification was achieved in a 50 μl reaction using Elongase Enzyme Mix (Invitrogen, Grand Island, NY), 100 ng DNA, and a thermal cycling protocol including an initial 30 s denaturation step at 94°C, 40 cycles of 94°C for 30 s, 64°C for 30 s, 68°C for 150 s, and a final elongation step at 72°C for 7 min.

**Table 2:** **Polymerase chain reaction (PCR) and sequencing primers used to amplify portions of the mtDNA and Y chromosome for phylogenetic analysis to verify Mauritius origin of the resequenced cynomolgus macaques from the Goswami et al.** [28] **study**

Gene	Primer Name	Sequence (5′ to 3′)
**mtDNA**		
HSVI; partial *cytb*		
	Forward	CCG CCC ACT CAG CCA ATT CCT GTT CT
	Reverse	CCC GTG ATC CAT CGA GAT GTC TT
**Y chromosome**		
*SRY*		
	SW2	*CTT GAG AAT GAA TAC ATT GTC AGG G*
	SW3B	*AGG TCT TTG TAG CCA ATG TTA CCC G*
	*F1*	*AGT GAA GCG ACC CAT GAA YG*
	*R1*	*GTA TCC CAG MTG CTT GCT GAT C*
*TSPY*		
	*TSPY-A*	*AGC CAG GAA GGC CTT TTC TCG*
	*TSPY5R*	*CTG TGC ATA AGA CCA TGC TGA G*
	*470 F*	*CGG CAG TTC TCT GCA T*
	*E690R*	*TCG RCA TGG ATA AGA CGG AC*

PCR product purification was performed using ExoSAP-IT (Affymetrix, Santa Clara, CA) and was outsourced to Functional Biosciences, Inc. (Madison, WI) for sequencing where Sanger sequencing reactions are performed using BigDye V3.1 on ABI 3730xl instruments. The PCR primers were used for sequencing the mtDNA region. Sequencing primers, however, were used from Tosi et al. [44] for the SRY gene (F1, R1) and for the TSPY gene (470 F, E690R) in addition to the PCR primers.

### Phylogenetic analysis

Sequence reads were assembled and cleaned-up using CodonCode Aligner v4.1.1 (CodonCode Corporation, Centerville, MA). Two alignments were created for phylogenetic analysis, one for the mtDNA sequence data and one for the Y chromosome data, using ClustalW [48]. Sequences from Tosi et al. [44] and Tosi and Coke [25] were included as references to establish provenance of our cynomolgus macaque samples and a single baboon (*Papio* sp.) sequence was included in each alignment as an outgroup for phylogenetic analysis. Each alignment was run in jModelTest v2.1.1 [49, 50] and Akaike information criterion (AIC) calculations were used to determine the best-fit model of nucleotide substitution for phylogenetic analysis. The model used for the Y chromosome dataset was the GTR + G model with alpha = 0.1450 based on model averaged estimates, and for the mtDNA dataset, the HKY + I + G with alpha = 0.3560 and I = 0.4194 based on model averaged estimates. Maximum likelihood phylogenetic analyses were carried out using PhyML 3.0 [51], with the best of nearest neighbor interchanges (NNI) and subtree pruning and regrafting (SPR) tree topology search, a BioNJ starting tree, and bootstrap analysis (n = 100).

### Polymorphism panel and genotyping

A panel of SNPs was generated to determine population substructure in a manner analogous to that which had been previously developed for the differentiation of Indian-origin and Chinese-origin rhesus macaques [32]. Our SNP selection strategy was aimed at minimizing the bias present in the original Goswami et al. [28] study. The original study suggested two subpopulations of cynomolgus macaques on Mauritius; however, multiple SNPs in a single linkage block could bias the results by potentially giving more weight to a single divergent position than is appropriate. To address this in this study we re-selected SNPs evenly across all 20 autosomes and at a distance that would preclude the possibility for two adjacent SNPs to be in the same linkage group. Selected SNPs were never less than 150 kb apart, with only 6 pairs less the 1 Mb apart, and a median distance between adjacent SNPs equaling 11 Mb.

We further selected SNPs based on their anticipated ability to differentiate between the two putative populations identified previously, prioritizing SNPs that displayed the greatest difference in minor allele frequencies (ΔMAF). This approach maximizes the information content of the SNPs and allows for the achievement of significant results with the use of fewer SNPs. Finally, any given SNP can be the result of demography or selection. We prioritized supposed neutral, demographic SNPs, but did not exclude SNPs possibly under selection as these too could be informative. However given that SNPs under selection may obscure signals of demography we prioritized non-genic SNPs followed by synonymous SNPs and then nonsynonymous SNPs. 110 SNPs were selected based on these criteria.

Assay design and SNP genotyping using Sequenom iPLEX technology were outsourced to the Biomedical Genomics Center at the University of Minnesota. Four assay pools (30, 28, 28, and 24 SNPs) were created for multiplexing. DNA samples were divided across three 96-well plates and duplicates were placed on each plate to ensure consistency in genotype calls. All DNA samples underwent quality control analysis prior to genotyping. 96 SNPs (divided evenly across the multiplexes) resulted in successful assays with >97% call rates and were used for future analyses (Additional file 7: Table S3). All duplicate individuals were called identically.

### Population analysis

Using the genotypes ascertained on the SNP panel, population substructure was interrogated using STRUCTURE 2.3.4. [35, 52]. STRUCTURE uses a Bayesian approach to identify subpopulation structure, returning a log probability (ln Pr (X|K) for the data for a given number of discrete clusters (K). For initial analyses, the default settings of STRUCTURE were used following the configuration of Falush et al. [53] with 10,000 burn-in and 40,000 Markov chain Monte Carlo repetitions. The degree of admixture, alpha, was allowed to be estimated from the data and a default value of lambda, a parameter describing the distribution of allele frequencies, was fixed. Allele frequencies were assumed to correlate between clusters. For between one (K = 1) and five clusters (K = 5), 100 runs each were tested. To test the robustness of these assumptions the same was also run with 50,000 burn-in and 250,000 MCMC repetitions, a data derived lambda (2.22), and assuming independence between allele frequencies in populations (Additional file 3: Figures S1, Additional file 4: Figure S2 and Additional file 5: Figure S3).

A Discriminant Analysis of Principle Components (DAPC) was also performed using the *adegenet* package v1.4-2 in R [37, 54–56]. DAPC uses a clustering algorithm *k*-means and Bayesian Inference Criterion to determine number of population clusters, K, optimizing variance between groups while minimizing variance within groups. SNP data was first transformed using a Principle Component Analysis (PCA) and then analyzed using k values from 1 to 10 with *k*-means to identify the optimal number of clusters. DAPC then constructs synthetic variables, discriminant functions, based on linear combinations of alleles harboring the greatest between-group variation and smallest within-group variation [37]. This method differs from traditional PCA analysis in that it minimizes within group variability.

### Availability of supporting data

All supporting data are included as additional files to this manuscript.

## Electronic supplementary material

Additional file 1: Table S1: Animals used in the mtDNA phylogenetic analysis to confirm that animals in the Goswami et al. [28] study derived from the island of Mauritius. GenBank accession numbers and reference information to previous work is also included. (XLSX 26 KB)

Additional file 2: Table S2: Animals used in the YDNA phylogenetic analysis to confirm that animals in the Goswami et al. [28] study derived from the island of Mauritius. GenBank accession numbers and reference information to previous work is also included. (XLSX 13 KB)

Additional file 3: Figure S1: Subpopulation inference after Evanno et al. [34], STRUCTURE model using λ = 2.22 with a 10 k burn-in and 40 k MCMC replicates. A. Mean ln (X|K) (±SD) over 100 runs for each value of K. B. Rate of change of ln (X|K) (mean ± SD). C. Absolute value of the second order rate of change (mean ± SD). D. ΔK, mean of ln“(X|K) divided by standard deviation of ln (X|K). The modal value is the presumptive true number of subpopulations. (PDF 13 KB)

Additional file 4: Figure S2: Subpopulation inference after Evanno et al. [34], default STRUCTURE model with a 50 k burn-in and 250 k MCMC replicates. A. Mean ln (X|K) (±SD) over 100 runs for each value of K. B. Rate of change of ln (X|K) (mean ± SD). C. Absolute value of the second order rate of change (mean ± SD). D. ΔK, mean of ln“(X|K) divided by standard deviation of ln (X|K). The modal value is the presumptive true number of subpopulations. (PDF 13 KB)

Additional file 5: Figure S3: Subpopulation inference after Evanno et al. [34], default STRUCTURE model with a 10 k burn-in and 40 k MCMC replicates, and subpopulation allele frequencies uncorrelated. A. Mean ln (X|K) (±SD) over 100 runs for each value of K. B. Rate of change of ln (X|K) (mean ± SD). C. Absolute value of the second order rate of change (mean ± SD). D. ΔK, mean of ln'“(X|K) divided by standard deviation of ln (X|K). The modal value is the presumptive true number of subpopulations. (PDF 13 KB)

Additional file 6: Figure S4: Inferred ancestry of individuals from STRUCTURE distinguishing animal source/dealer. Inferred ancestry with K = 2. Animals in red are derived from Cynologics and animals in blue are from Bioculture Mauritius. (PDF 37 KB)

Additional file 7: Table S3: SNPs included on the panel of 96 used to evaluate population substructure of cynomolgus macaques on Mauritius. Table also includes SNP location information including chromosome number and position, gene, gene region, and the flanking sequence. (XLSX 18 KB)
