# Supplementary figures and images for: Genetic substructure in cynomolgus macaques (Macaca fascicularis) on the island of Mauritius
Source: BMC Genomics. 2014 Aug 31;15(1):748. doi: 10.1186/1471-2164-15-748 (PMC4167525; doi:10.1186/1471-2164-15-748)

A.  $\ln \Pr(X|K)$

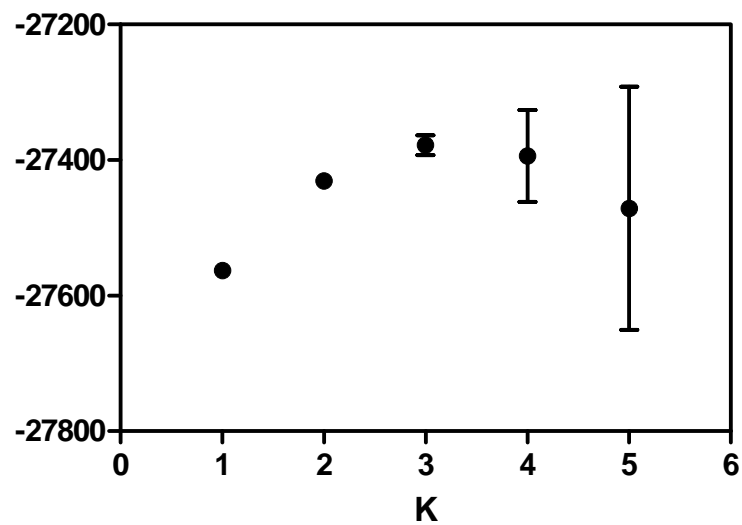

B.  $\ln' \Pr(X|K)$

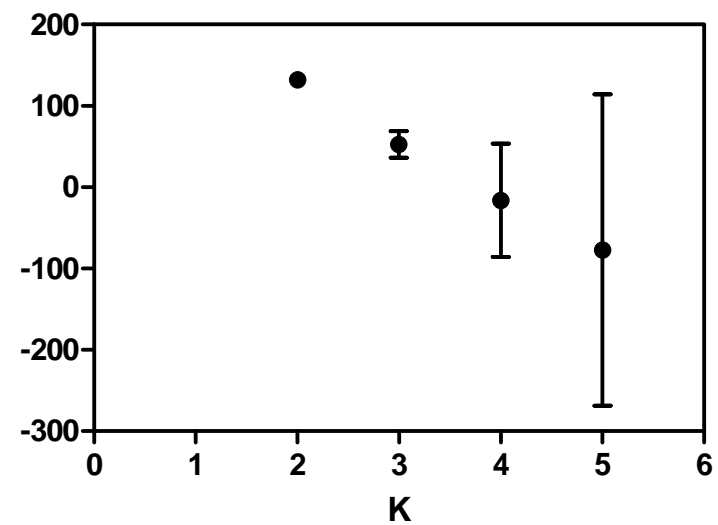

C.  $|\ln'' \Pr(X|K)|$

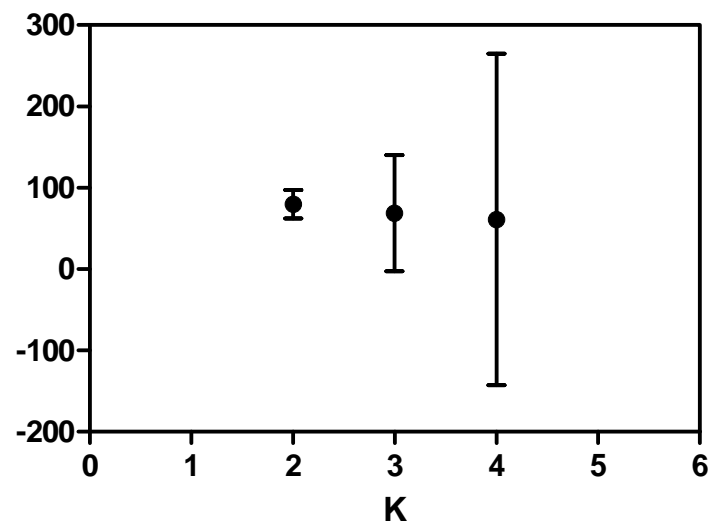

D.  $\Delta K$

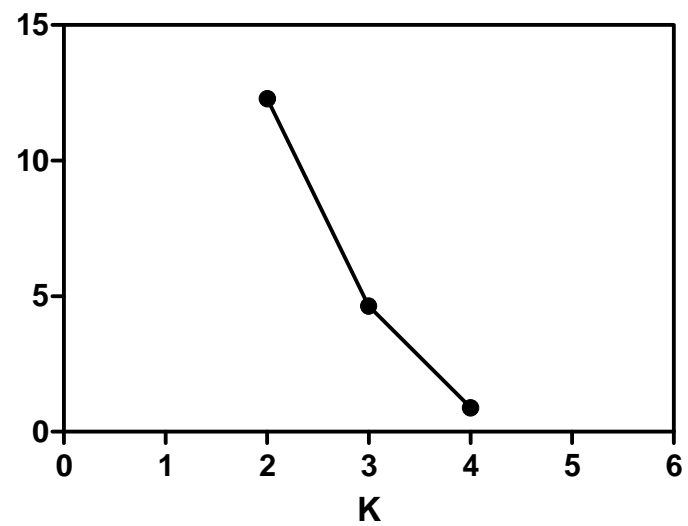

Supplement: Supplementary file 3 — Additional file 3: Figure S1: Subpopulation inference after Evanno et al. [34], STRUCTURE model using λ = 2.22 with a 10 k burn-in and 40 k MCMC replicates. A. Mean ln (X|K) (±SD) over 100 runs for each value of K. B. Rate of change of ln (X|K) (mean ± SD). C. Absolute value of the second order rate of change (mean ± SD). D. ΔK, mean of ln“(X|K) divided by standard deviation of ln (X|K). The modal value is the presumptive true number of subpopulations. (PDF 13 KB) [file 12864_2014_6440_MOESM3_ESM.pdf]

A.  $\ln \Pr(X|K)$

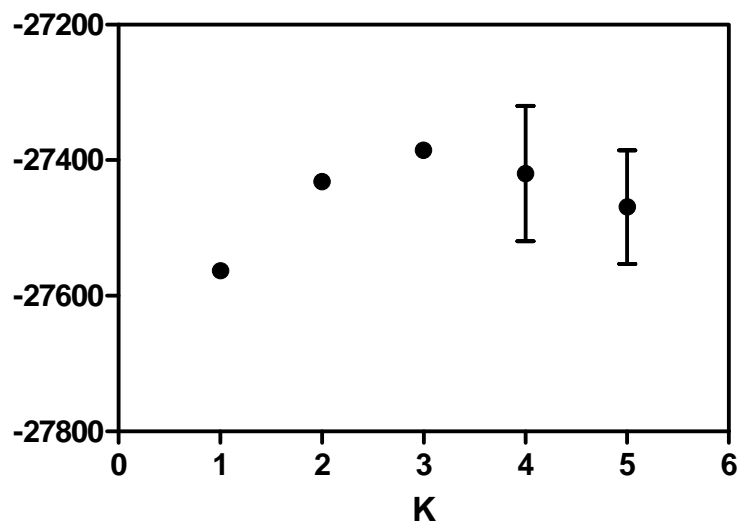

B.  $\ln' \Pr(X|K)$

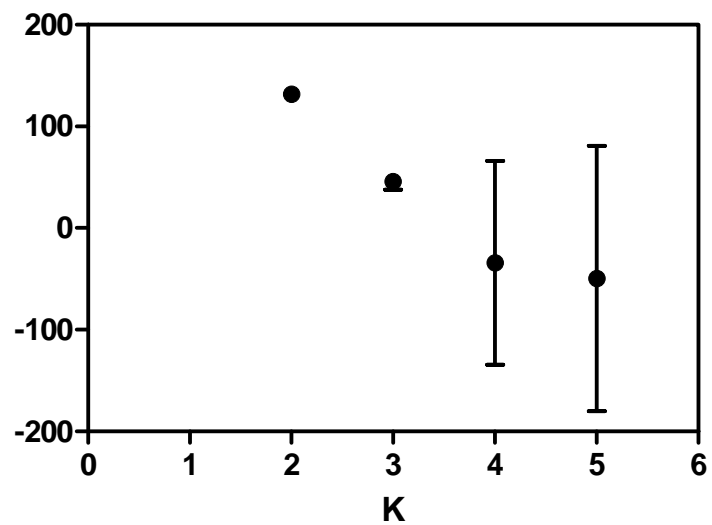

C.  $|\ln'' \Pr(X|K)|$

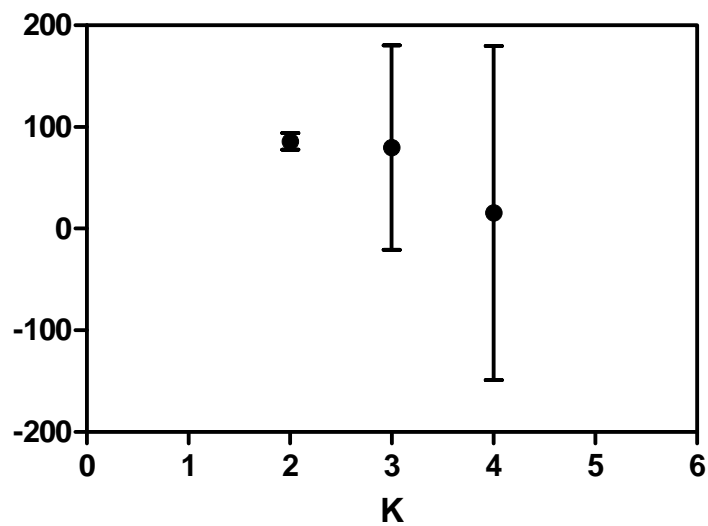

D.  $\Delta K$

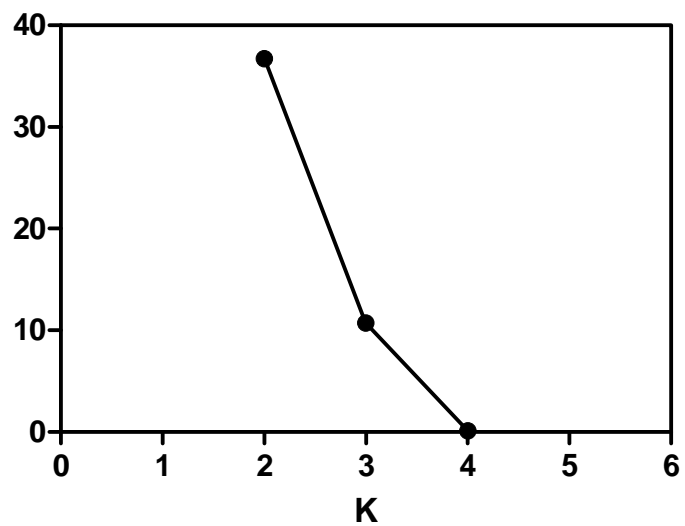

Supplement: Supplementary file 4 — Additional file 4: Figure S2: Subpopulation inference after Evanno et al. [34], default STRUCTURE model with a 50 k burn-in and 250 k MCMC replicates. A. Mean ln (X|K) (±SD) over 100 runs for each value of K. B. Rate of change of ln (X|K) (mean ± SD). C. Absolute value of the second order rate of change (mean ± SD). D. ΔK, mean of ln“(X|K) divided by standard deviation of ln (X|K). The modal value is the presumptive true number of subpopulations. (PDF 13 KB) [file 12864_2014_6440_MOESM4_ESM.pdf]

A.  $\ln \Pr(X|K)$

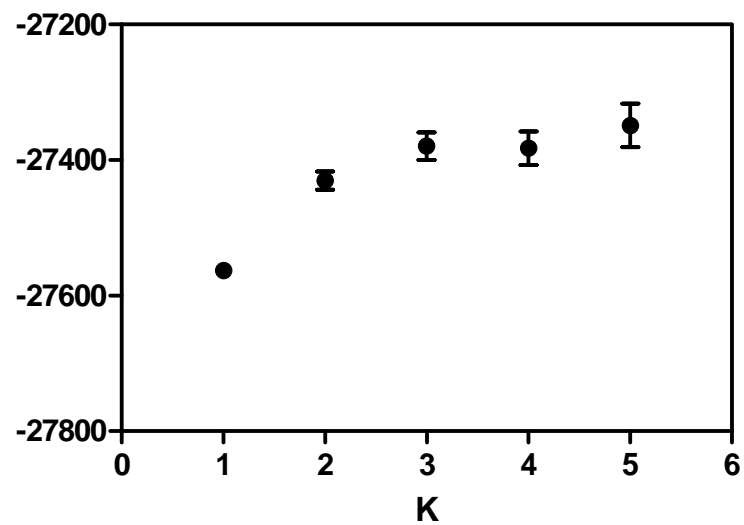

B.  $\ln' \Pr(X|K)$

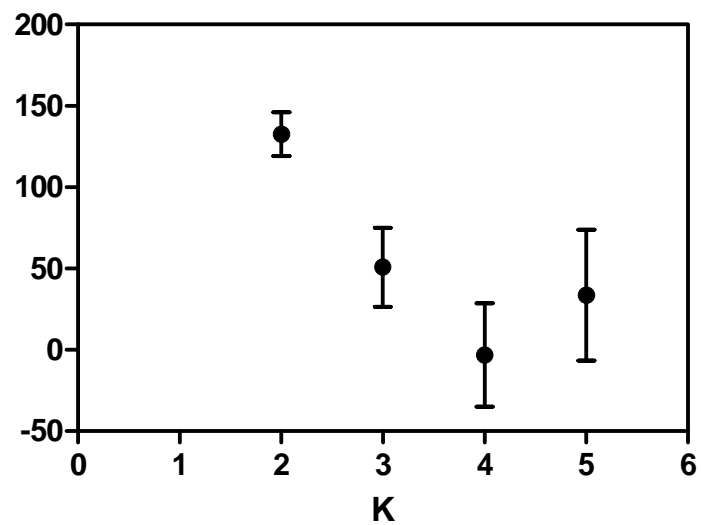

C.  $|\ln'' \Pr(X|K)|$

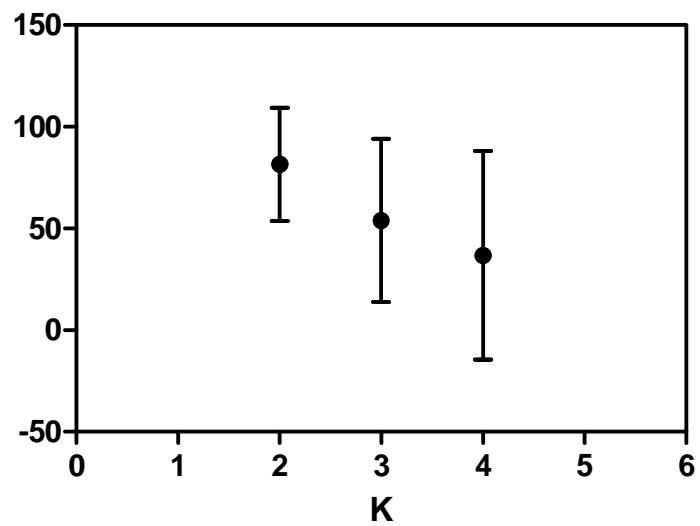

D.  $\Delta K$

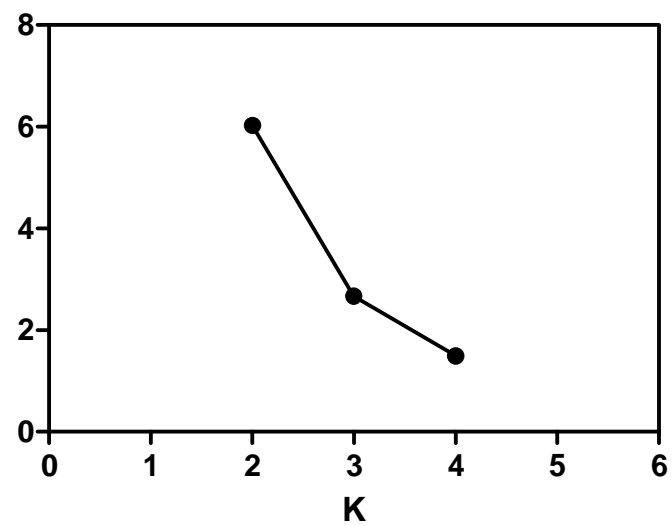

Supplement: Supplementary file 5 — Additional file 5: Figure S3: Subpopulation inference after Evanno et al. [34], default STRUCTURE model with a 10 k burn-in and 40 k MCMC replicates, and subpopulation allele frequencies uncorrelated. A. Mean ln (X|K) (±SD) over 100 runs for each value of K. B. Rate of change of ln (X|K) (mean ± SD). C. Absolute value of the second order rate of change (mean ± SD). D. ΔK, mean of ln'“(X|K) divided by standard deviation of ln (X|K). The modal value is the presumptive true number of subpopulations. (PDF 13 KB) [file 12864_2014_6440_MOESM5_ESM.pdf]

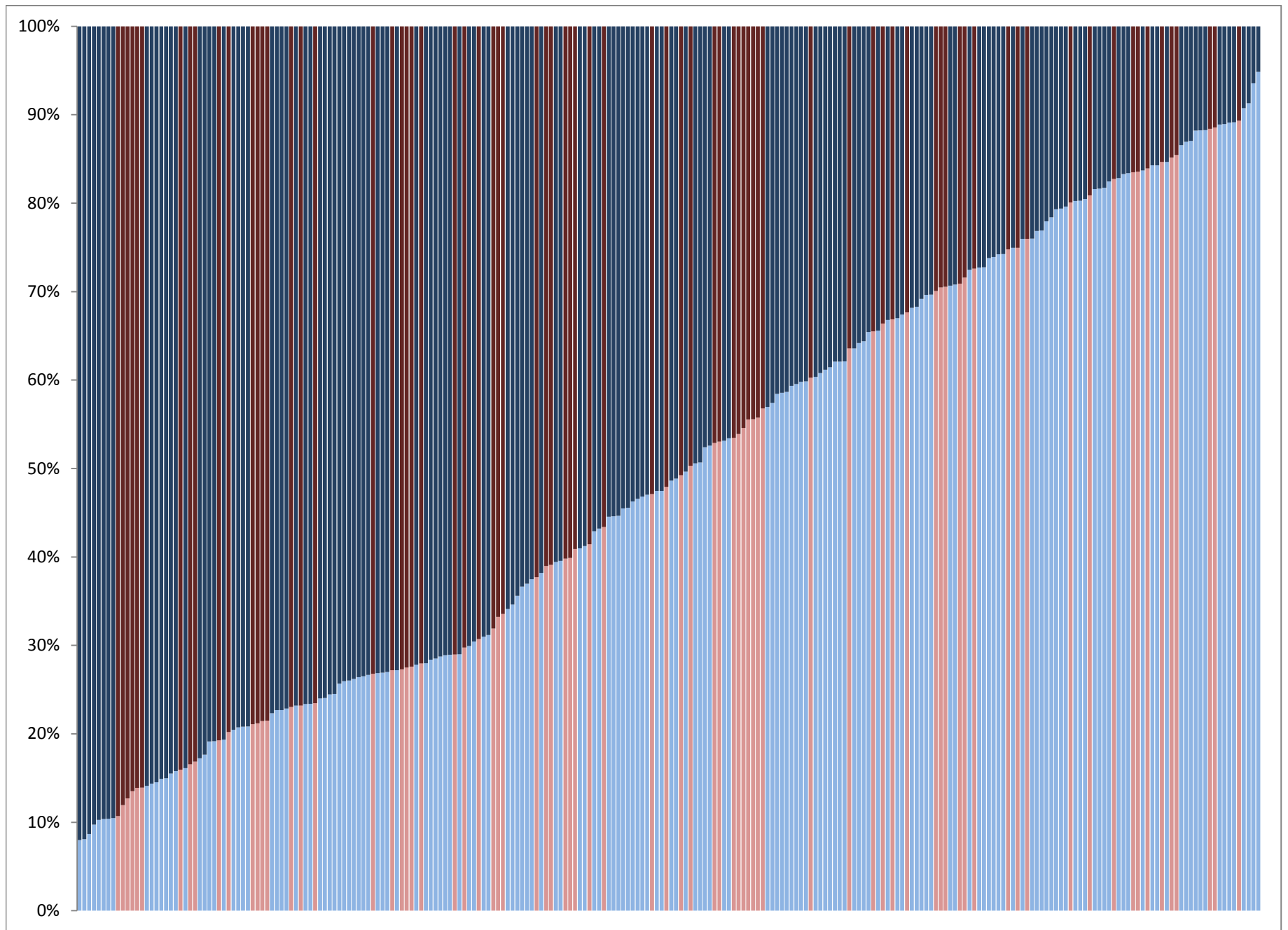

Supplement: Supplementary file 6 — Additional file 6: Figure S4: Inferred ancestry of individuals from STRUCTURE distinguishing animal source/dealer. Inferred ancestry with K = 2. Animals in red are derived from Cynologics and animals in blue are from Bioculture Mauritius. (PDF 37 KB) [file 12864_2014_6440_MOESM6_ESM.pdf]
